# Rickettsial retinitis—an Indian perspective

**DOI:** 10.1186/s12348-015-0066-8

**Published:** 2015-11-26

**Authors:** Ankush Kawali, Padmamalini Mahendradas, Priya Srinivasan, Naresh Kumar Yadav, Kavitha Avadhani, Kanav Gupta, Rohit Shetty

**Affiliations:** Uveitis and Ocular Immunology Services, Narayana Nethralaya, 121/C, Chord Road, 1st ‘R’ Block, Rajajinagar, Bangalore, 560010 India; Vitreo-retinal Services, Narayana Nethralaya, Bangalore, India; Cornea and Refractive Services, Narayana Nethralaya, Bangalore, India

**Keywords:** Rickettsia, Retinitis, India, Uveitis, Typhus

## Abstract

**Background:**

Though rickettsiosis is common in India, there is paucity of rickettsial retinitis (RR) reports from India. Moreover, rickettsial sub-types and their association with retinitis have not been studied. We are reporting a case series of presumed RR with their course of the disease, visual outcome, and association with rickettsial sub-type based on Weil-Felix test.

**Findings:**

This is a retrospective study of 19 eyes of 10 patients presented to a single institution. Cases diagnosed with presumed RR were identified from our database from March 2006 to October 2014 and studied retrospectively for patient’s demography, clinical presentation, and treatment. Patients with history of fever, retinitis, and a positive Weil-Felix test and a negative chikungunya and dengue serology were diagnosed as presumed rickettsial uveitis. One patient was diagnosed to have epidemic typhus, and four were diagnosed to have Indian tick typhus. Nine patients had bilateral presentation. One patient had history of dog tick bite, and four patients had skin rashes. All the patients presented between 2 and 4 weeks after a fever.

**Conclusions:**

Retinitis on posterior pole with recent history of fever with or without skin rash and a positive Weil-Felix test may suggest a rickettsial etiology. Its ocular manifestation could be an immune response to recent systemic rickettsial infection. Indian tick typhus and epidemic typhus could be the common sub-types seen in our population. Although it has aggressive presentation, it has a good visual prognosis.

## Findings

### Introduction

Rickettsial zoonoses are caused by obligate intracellular gram-negative bacteria. Most of them are transmitted to humans by the bite of contaminated arthropods, such as ticks and mites. Rickettsial agents are classified into three major categories: the spotted fever group, the typhus group, and the scrub typhus, although later has been re-classified as a new genus—*Orientia*. Rickettsial diseases are characterized by fever, malaise, and skin rash. There are several reports of ocular involvement in rickettsiosis worldwide [1–3]. However, in spite of many systemic rickettsial reports, no ocular manifestations of rickettsiosis have been reported from India [4, 5]. Moreover, rickettsial sub-classification and their association with retinitis remain unknown. Herein, we report demography, posterior segment manifestations, visual outcome, and association with rickettsial sub-type of the disease.

### Subjects and methods

**Fig. 1:**
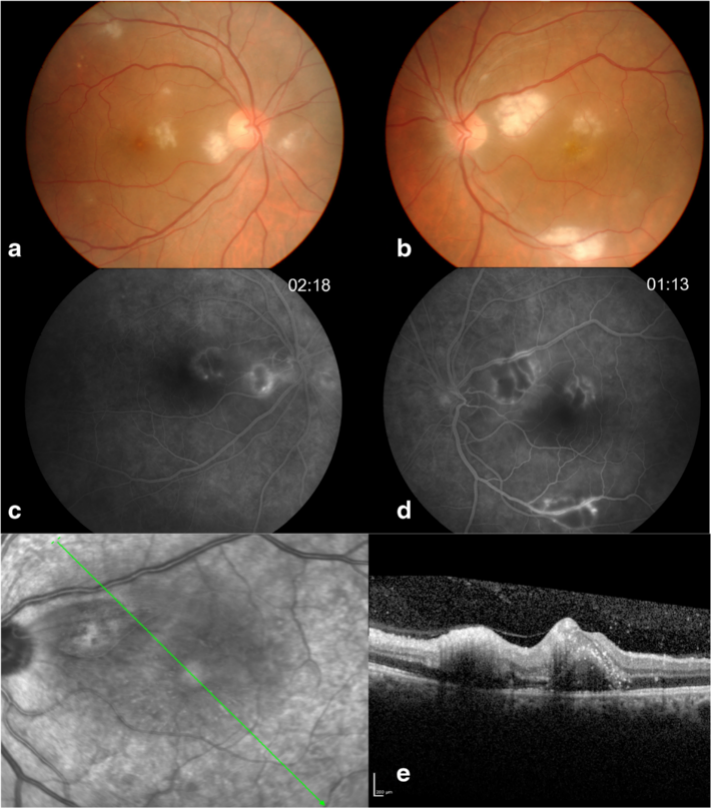
A 59/M with history of travel to north India (Delhi) presented with multifocal retinitis with macular edema (**a**, **b**). Early hypofluorescence and late hyperfluorescence at the borders of retinitis lesions and minimal vascular staining and leakage was noted on FFA (**c**, **d**). OCT revealed inner retinal hyperreflectivity, intraretinal hyperreflective dots and shallow sub-retinal fluid (**e**)

All cases diagnosed as presumed rickettsial retinitis (RR) on the basis of positive Weil-Felix test (WFT) from March 2006 to October 2014 were identified from our database. Patient’s demographics, history of present illness, clinical findings, investigations, and treatment details were studied. Narayana Nethralaya Institutional review board and ethical committee approval was obtained for this study. Cases presented with focal or multifocal retinitis at posterior pole (Fig. 1a, b) with or without spill over inflammation into the anterior segment and with a history of fever and a positive WFT were diagnosed as presumed RR. Weil-Felix kit was used from Plasmatec UK, and titers 1:160 and more were considered positive (Table 1). WFT interpretation and rickettsial sub-classification was done according to Table 2. All patients had a negative chikungunya and dengue serology and a negative human immunodeficiency virus (HIV) tri-dot test. Of 10 patients, 19 eyes were studied retrospectively for their demography, clinical presentation, treatment, visual outcome, and type of rickettsial disease. Immunofluorescence assay (IFA), polymerase chain reaction (PCR), and rapid immunochromatographic test (RICT) for rickettsia were not done due to its unavailability in our setup.

**Table 1:** Investigations and molecular diagnostic done to identify etiology

Sr. no.	Rickettsial sub-classification	Weil-Felix test			Chikungunya serology	Dengue serology	WNV and JE serology	MP in blood film	WIDAL	PCR for scrub typhus
		OX 2	OX K	OX 19						
1	Unclassified	1:1280	Negative	1:40	Negative	Negative	Not done	Negative	Negative	Not done
2	ET	Negative	Negative	1:160	Negative	Negative	Negative	Negative	Negative	Negative
3	Unclassified	1:160	1:160	1:320	Negative	Negative	Not done	Negative	Negative	Not done
4	ITT	1:160	Negative	1: 160	Negative	Negative	Not done	Negative	Negative	Not done
5	Unclassified	1:160	1:80	Negative	Negative	Negative	Negative	Negative	Not done	Negative
6	Unclassified	1:320	1: 40	1:80	Negative	Negative	Not done	Not done	Not done	Not done
7	Unclassified	1:160	1: 160	1:160	Negative	Negative	Negative	Not done	Negative	Negative
8	ITT	1: 320	Negative	1: 320	Negative	Negative	Not done	Not done	Not done	Not done
9	ITT	1:320	Negative	1:160	Negative	Negative	Not done	Negative	Negative	Not done
10	ITT	1:640	Negative	1:160	Negative	Negative	Not done	Negative	Negative	Not done

*WNV* West Nile virus, *JE* Japanese encephalitis, *MP* malaria parasite, *WIDAL* typhoid, *ITT* Indian tick typhus, *ET* epidemic/endemic typhus

**Table 2:** Weil-Felix test interpretation

OX 2	OX K	OX 19	Rickettsial sub-classification
+	−	+	Indian tick typhus (spotted fever)
−	−	+	Epidemic/endemic typhus
−	+	−	Scrub typhus

**Table 3:** Demography, systemic, and ocular presentation and treatment

Sr. no.	Age/sex		Fever			BCVA at presentation		BCVA at the final follow-up		Anterior segment	Posterior segment	Antibiotics	Steroids
			Skin rash	Joint pain	Ocular involvement								
						OD	OS	OD	OS				
1	F	40			27 days	20/60	20/40	20/20	20/20	OU: quiet, AVF: cells+	OU: MFR, M.S.	Doxy	Oral
2	M	27			10 days	20/2000	20/20	20/40	20/20	OS: O.5 cells, AVF: cells+	RE: MFR	Doxy	Oral + OD PST
3	M	16		√	10 days	20/2000	20/80	20/60	20/30	OU: quiet, AVF: cells+	OU: MFR hemorrhages, M.E.	Nil	Oral
4	F	33			21 days	20/2000	20/60	20/30	20/30	OU: quiet, AVF: cells++	OU: MFR, M.E.	Doxy	Oral + OS PST
5	F	23			30 days	20/125	20/2000	20/60	20/60	OU: cells 1 +, AVF: Cells+	OU: MFR, M.E.	Nil	Oral + OU PST
6	M	29	√	√	15 days	20/30	20/20	Lost to follow-up		OD: quiet, AVF: cells+	OD: focal retinitis	Doxy	Oral
7	F	16	√	√	28 days	20/2000	20/800	20/30	20/30	OU: N.G. KPs, cells+, AVF: cells++	OU: MFR, M.E.	Doxy	IVMP + Oral
8	F	34	√		15 days	20/30	20/20	20/20	20/20	OU: quiet, AVF: cells+	OU: MFR	Doxy	Oral + OS PST
9	M	64			27 days	20/80	20/2000	20/30	20/40	OU: N.G. KPs, cells+, AVF: cells+	OU: MFR, M.E.	Doxy	Oral + OD PST
10	F	22	√		30 days	20/6000	20/20	Lost to follow-up		OU: cells+, AVF: cells+	OU: MFR, OD: M.E., D.E.	Nil	Oral

*AVF* anterior vitreous face, *MFR* multifocal retinitis, *M.E.* macular edema, *D.E.* disc edema, *M.S.* macular star, *PST* posterior sub-Tenon’s injection of Triamcinolone, *IVMP* intravenous methylprednisolone, *Doxy* doxycycline

### Results

**Fig. 2:**
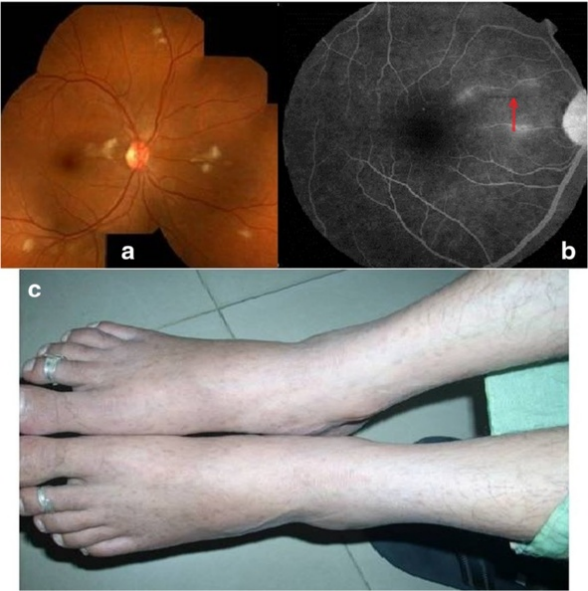
34/F, fundus photograph at presentation showed multifocal retinitis patches (**a**). FFA revealed a small occluded arteriole (*arrow mark*) (**b**). Resolving macular rashes on both the feet at the presentation (**c**)

**Fig. 3:**
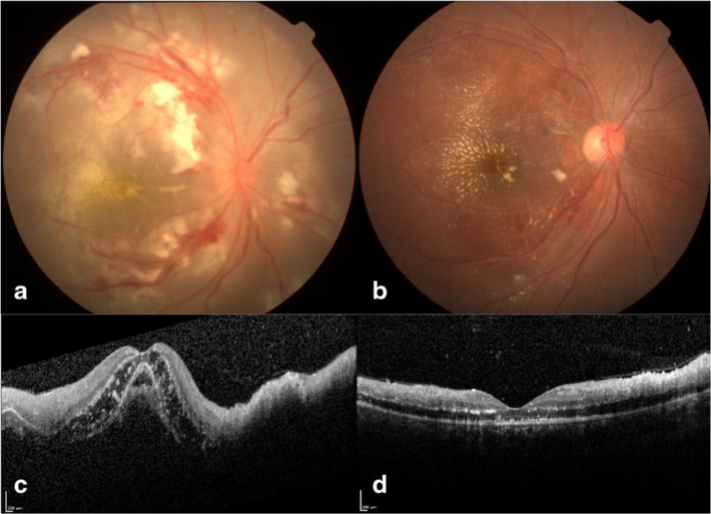
16/F, fundus at presentation showed disc edema, multifocal retinitis patches along with hemorrhages, and macular edema with early macular fan appearance (**a**). and at the final follow-up (after 2 months), resolved disc edema, retinitis, hemorrhages, and macular edema with macular star formation (**b**) after being treated with IVMP followed by oral steroids and oral doxycycline. OCT scan showed inner retinal hyperreflectivity, intraretinal hyperreflective dots, and gross sub-retinal fluid on OCT (**c**). Note the resolved sub-retinal fluid, resolving inner retinal thickening, condensation of intraretinal exudates, and resolved macular edema (after 2 weeks) (d)

Nineteen eyes of 10 patients were studied.(Table 3) Mean age of presentation was 30.4 years (range 16–64). Four patients were males, and six patients were females. Only one patient (case 6) had unilateral presentation. All patients had history of fever 10 days to 1 month prior to presentation. Four patients had skin rash (Fig. 2c), and three had history of joint pain along with the fever. Case 6 was bitten by a dog tick following which he developed a skin rash on his back and subsequently fever. All the patients were presented with sudden onset of visual loss with mild to moderate redness and pain. Mean presenting visual acuity was 20/60 (range, 20/20–20/2000). Anterior chamber reaction was mild to moderate with nongranulomatous keratic precipitates in four eyes. All patients had focal (*n* = 1, case 6) or multifocal (*n* = 9) cotton wool spots like lesions of size ranged from ½ disc diameter (DD) to 3 DD suggestive of patchy retinitis on the posterior pole and around the disc, with or without disc edema (*n* = 1) or macular edema (*n* = 13). (Fig. 1a, b) In six cases, few hemorrhages along with retinitis patches were also seen. Vitritis was mild to moderate in all affected eyes. None of the patients had grade 3 or grade 4 vitritis. No obvious retinal vascular sheathing was observed clinically. In all cases, retinitis lesions were resolved within 3–4 months after treatment. (Fig. 3a, b). Fundus fluorescein angiography (FFA) was done in three cases, which revealed early hypofluorescence corresponding to retinitis patches which gradually turned hyperfluorescent at the border of the retinitis lesions. Mild leakage from adjacent vessels was also noted (Fig. 1c, d); another case showed disc leakage and peripheral capillary leakage in addition to the above findings. Case 8 showed minor arteriolar occlusion on FFA (Fig. 2b). Optical coherence tomography (OCT) showed increased inner retinal reflectivity after shadowing corresponding to the area of retinitis and confirmed serous macular detachment in 11 eyes (57.89 %) (Fig. 1e). Titers for WFT were as shown in Table 1. In two patients (cases 7 and 9), WFT was repeated at the final follow-up (after 4–5 months of the fever) which showed decreasing titers. Four patients were diagnosed as Indian tick typhus (ITT), one as epidemic typhus (ET), and in the rest of the patients, rickettsia sub-classification remained ambiguous. Serology for chikungunya and dengue showed negative results in all. Three patients were also evaluated for West Nile virus (WNV) and Japanese encephalitis serology and were tested negative for same. Anterior chamber tap for scrub typhus PCR was negative in these three patients. None of the patients were immunocompromised, and all tested negative for HIV tri-dot test and ANA. Patient with focal retinitis (case 6) was negative for toxoplasma serology. Seven patients received oral doxyclycline (200 mg/day) for 2 weeks and three were managed with only steroids and dosage that were titrated to the severity of inflammation. One patient received i.v. methylprednisolone, and six eyes received posterior sub-Tenon’s injection of triamcinolon acetonide (PST). All the patients had good resolution of the inflammation and improvement in vision. Mean final visual acuity was 20/30 (range, 20/20–20/60). Two patients were lost to follow-up. Mean follow-up duration was 2.5 months.

### Discussion

Focal or multifocal retinitis post febrile illness is known to be caused by chikungunya, dengue, West Nile virus (WNV), bartonellosis, Lyme’s disease, rift valley fever, etc. Rickettsial retinitis also contributes significant portion of this condition. Posterior segment manifestations in Mediterranean spotted fever are well studied by Khairallah et al. [2]. A report from Japan noted conjunctival congestion, conjunctival hemorrhages, and dilatation of episcleral vessels in scrub typhus [1]. Parola et al. reported a case of Indian tick typhus in a French traveler after her stay in India who had conjunctivitis and disturbance of vision [6]. Studies from India on scrub typhus have not reported ocular involvement [7, 8]. A case series of *Rickettsia conorii* from Tunisia has reported rickettsial infection in spring or summer, whereas scrub typhus outbreak has been reported in cooler months in India [9]. All our patients presented in relatively colder months of the year (September–March). This suggests that RR is an epidemic which repeats every year in our population. The relationship between *R. conorii* and brown dog tick has been studied, and they have noted that the change in temperature and physiology of the tick host induces the organism to emerge from dormancy and attain infectivity [10]. Recall of insect bite (a dog tick) was elicited only in one patient in our series. It is possible that the skin rash (Fig. 2) being masked in dark-skinned individuals was easily missed by the patients or physicians. The value of WFT, though not a gold standard, has been proven in Indian studies [4, 7]. In a study from Karnataka, India, WFT titers more than 1:160 for OX K and more than 1:80 for OX 2 and OX 19 that were considered significant [4]. In our study, these titers were more for OX 2 and OX 19 compared to their study. Similar to Ajantha et al.’s study [4], demonstration of fourfold rise of the titer of Weil-Felix in the convalescent serum was not possible in our patients as they presented to us after 2 to 4 weeks of the fever and WFT was not done during their fever, but decreasing titers of OX 2 and OX 19 was noticed in two of our patients (cases 7 and 9) when the test was repeated 3 and 4 months after the presentation (i.e., after 4–5 months of the fever). Visual impairment was significant in most of our cases due to macular involvement. All our cases presented with nongranulomatous panuveitis or posterior uveitis with retinitis as a common feature. Though vascular sheathing was not obvious clinically in all cases, FFA in two cases revealed vascular staining and minimal leakage from affected vessels and a minor arteriolar occlusion was seen in case 8 on FFA (Fig. 2b). Most of these findings were consistent with the findings described by Khairallah et al [2]. Unlike Kahloun et al.’s report, retinal vascular occlusion was not noted clinically in our case series [3]. This could be because of small number of cases and exclusion of cases without retinitis in our case series. Six eyes with severe macular involvement received PST and showed remarkable improvement. Patient who received only steroids improved equally compared to the patients who received antibiotics and steroids. Kahloun et al. have also described a patient who showed spontaneous improvement (without antibiotics) while serological test was pending [3]. Though rickettsial endogenous endophthalmitis has been reported [11], bilaterality in 9 out of 10 cases, an interval of 2 to 4 weeks between the systemic and ocular presentation and better response to 'steroid-only' treatment in 3 patients in our study points towards an immune-driven process. No patient was found to have scrub typhus in our series based on WFT as well as by PCR of ocular fluid in three cases. No report of retinitis was documented even in larger studies on scrub typhus from India [9]. Occurrence of retinitis in scrub typhus is probably rare in Indian sub-continent.

We suggest the following diagnostic approach for patients with retinitis with a history of recent fever in Indian scenario: After ruling out hypertensive and diabetic retinopathy, a baseline blood investigations including total and differential counts, erythrocyte sedimentation rate (ESR), TPHA or rapid plasma reagin (RPR), HIV, anti-nuclear antibody (ANA), and toxoplasma serology should be done. Other serological investigations to know the cause of the fever can be considered depending on the clinical features, availability, and affordability: chikungunya, dengue, West Nile virus, WFT, and/or indirect immunofluorescent or RICT. One can also consider ocular fluid analysis and PCR for cytomegalovirus (CMV), herpes simplex virus, varicella zoster virus (VZV), and serology for *B. henselae* if poor response to steroid treatment is observed to rule out atypical presentation of the above entities; FFA helps to detect vasculitis component and its severity but OCT is highly recommended as it shows inner retinal involvement in epidemic retinitis including RR which can differentiate retinitis due to other etiologies such as toxoplasmosis, CMV, or VZV which generally involve full thickness of the retina [12]. Its usefulness in monitoring response to the treatment is well-known. Once the patients are diagnosed as RR or presumed RR, they can be started on steroids and antibiotics. The role of antibiotics in RR is unclear [3]. In our study, only three patients were treated with 'steroid-only' regimen for retinitis but they received antibiotics during their fever. Larger randomized control studies are needed to study recovery speed in RR with steroids alone versus steroid-antibiotics combination.

Our study suggests that presumed RR is an epidemic, and its ocular manifestation could be an immune response to recent systemic rickettsial infection. Indian tick typhus and epidemic typhus could be common causes of RR as compared to scrub typhus in India. To the best of our knowledge, this is the first case series on RR from India.

## Abbreviations

ANA: anti-nuclear antibody
CMV: cytomegalovirus
ESR: erythrocyte sedimentation rate
FFA: fundus fluorescein angiography
HIV: human immunodeficiency virus
IFA: immunofluorescence assay
IVMP: intravenous methylprednisolone
OCT: optical coherence tomography
PCR: polymerase chain reaction
RICT: rapid immunochromatographic test
RPR: rapid plasma reagin
RR: rickettsial retinitis
VZV: varicella zoster virus
WFT: Weil-Felix test
WNV: West Nile virus
PST: Posterior Sub-tenon's injection of triamcinolone
